# Quality of sleep, health and well-being in a population-based study

**DOI:** 10.11606/s1518-8787.2019053001067

**Published:** 2019-09-18

**Authors:** Marilisa Berti de Azevedo Barros, Margareth Guimarães Lima, Maria Filomena Ceolim, Edilson Zancanella, Tânia Aparecida Marchiori de Oliveira Cardoso

**Affiliations:** I Universidade Estadual de Campinas. Faculdade de Ciências Médicas. Departamento de Saúde de Coletiva. Campinas, São Paulo, Brasil; II Universidade Estadual de Campinas. Faculdade de Enfermagem. Campinas, São Paulo, Brasil; III Universidade Estadual de Campinas. Faculdade de Ciências Médicas. Departamento de Oftalmologia/otorrinolaringologia. Campinas, São Paulo, Brasil; IV Universidade Estadual de Campinas. Faculdade de Ciências Médicas. Departamento de Neurologia. Campinas, São Paulo, Brasil

**Keywords:** Sleep, Comorbidity, Risk Factors, Socioeconomic Factors, Mental Health, Health Surveys

## Abstract

**OBJECTIVE:**

To estimate the prevalence of poor self-rated sleep and to identify the population subgroups most susceptible to the problem.

**METHODS:**

This is a cross-sectional, population-based study developed with data from the Health Survey conducted in the city of Campinas (ISACamp 2014/2015). Data from a sample of 1,998 individuals aged 20 years or older were analyzed. The self-rated quality of sleep was analyzed according to socio-demographic characteristics, morbidities, health behaviors and feeling of well-being. The association of sleep quality with different complaints and characteristics of sleep was also analyzed. Adjusted prevalence ratios were estimed using Poisson multiple regression model allowing for the sample weights.

**RESULTS:**

Prevalence of poor self-rated sleep was 29.1% and showed to be significantly higher in women, in individuals aged from 40 to 50 years, migrants, without occupation, physically inactive in leisure context, with common mental disorder (PR = 1.59), with greater number of health problems (PR = 2.33), poor self-rated health (PR = 1.61), and life dissatisfaction. Poor sleep was strongly associated with reports of difficulty in initiating sleep (PR = 4.17), in maintaining sleep (PR = 4.40) and with never or almost never feeling well when waking up (PR = 4.52).

**CONCLUSIONS:**

The results identify the population subgroups with poor quality of sleep that deserve greater attention. It also highlight the need to consider, in addition to the presence of comorbidities, mental health and the feeling of well-being in the care of patients with sleep problems and in the interventions planed for promoting healthy sleep.

## INTRODUCTION

Quality of sleep is one of the five factors considered relevant for the assessment of healthy sleep, understood as a multidimensional pattern of sleep-wake adapted to individual, social and environmental demands and that provides physical and mental well-being^1^ . As a basic human necessity, sleep is one of the most relevant emerging themes, because there is strong evidence that sleep deprivation and sleep disturbances affect metabolic and inflammatory processes, with broad negative impacts on health^2^ .

Specifically regarding sleep quality, research has detected that the worst quality is associated with higher mortality rates, prevalence of metabolic syndrome, diabetes, hypertension, coronary disease, and depression^3^ .

Sleep disorders, which imply low sleep quality, are also frequent causes of traffic and work accidents^7^ resulting from excessive daytime sleepiness. Low quality of sleep causes losses in the daily activities of the individual, affecting the productivity at work and the quality of life in general, which causes a strong social and economic impact^2^ .^.^

Sleep quality can be assessed by different instruments, in particular by the Pittsburgh Sleep Quality Index (PSQI)^8^ . One item of this instrument is on the individual subjective perception of sleep quality, and some authors have used a single question to perform this evaluation^9^ . Similarly, in the field of health assessment, information obtained by a single question about self-evaluated health proved to be highly correlated with mortality, diseases and other outcomes, becoming one of the most used health indicators nowadays^13 , 14^ .

Due to the strong impact of sleep quality on populations’ health, their patterns and trends must be monitored to identify the most vulnerable social and demographic segments, signaling appropriate strategies of control and treatment of disorders and initiatives to promote healthy sleep. However, of the six Brazilian population-based surveys that investigated sleep characteristics in adults^15^ , only one article was published focusing specifically on the adult sleep quality and the associated factors^17^ , in which only individuals aged 40 years or more participated.

From this perspective, considering the lack of studies on sleep quality, the observed growth of sleep problems and disorders^18^ , its impact on health and quality of life and the growing relevance of the theme in the field of public health, the aim of this study was to analyze the epidemiological profile of poor self-rated sleep according to a broad set of characteristics, including social and demographic, health status, morbidities, health behaviors and indicators of well-being. The aim of this research is also to analyze the association of self-perceived sleep quality with the report of complaints and characteristics of sleep in adults.

## METHODS

This is a cross-sectional, population-based study developed with data from the Health Survey of the city of Campinas (ISACamp 2014/15). This inquiry was conducted in a representative sample of the population residing in private and permanent households in the urban area of the city of Campinas, SP, Brazil. The objective of the survey was to collected data on health conditions and use of health services for people aged 10 years or older and then three age domains were defined – adolescents, adults and older adults – from which independent samples were drawn.

A minimum sample size of 1,000 people was defined for adolescents and older adults and 1,400 for adults. These sample sizes allow estimating a proportion of 0.50 (maximum sample variability), with sampling error of up to 5 percentage points, with a confidence level of 95% and considering a design effect of 2. The inquiry was conducted in a sample obtained by a stratified probabilistic cluster sampling developed in two stages. In the first stage, 70 census tracts were drawn, for which the updated list of households was made. To obtain the minimum size of individuals in each age domain and based on the average number of residents of each domain per household, 3,119 households were randomly selected to interview adolescents, 1,029 for adults and 3,157 for older adults, considering non-response rates of 27%, 22% and 20% for their age domains. All individuals in the domain for whom the residence had been drawn were interviewed. In this study, only data from individuals aged 20 years or older were analyzed.

The ISACamp 2014/15 questionnaire covers broad health themes and, in this study, information about sleep, lifestyle, morbidities, health status, emotional health, health behaviors, and demographic and socioeconomic conditions were analyzed.

Home interviews were conducted by trained interviewers, who used a software specially developed for this research and applied through a Samsung Galaxy, model GT-P5200 tablet.

The dependent variable of the study was self-assessment of sleep, constructed from the question “How do you evaluate the quality of your sleep? Would you say that it is: excellent/very good; good; regular; poor; very poor ?” We grouped the alternatives excellent/very good and good, in the category of good sleep; the regular, poor and very poor answers comprised the category of poor self-rated sleep.

The independent variables analyzed were:

- Demographic and socioeconomic: sex, age, marital status, place of birth, schooling in years of study, per capita family income, working condition and number of residents.
- Variables of health behaviors: weekly frequency and risk consumption of alcoholic beverage, assessed by AUDIT with cutoff point of 8 or more^19^ ; smoking: smoker, former smoker, never smoked; physical activity (PA) in leisure context, obtained with the International Physical Activity Questionnaire (IPAQ) and classified in active: 150 minutes of moderate PA or 75 minutes of vigorous PA; insufficiently active: when not reached the active level; and inactive: without PA practice.
- Morbidity and health status: number of chronic diseases among those reported as diagnosed by healthcare professional and included in the checklist (hypertension, diabetes, angina/infarction, cancer, asthma, rhinitis, sinusitis, arthritis /rheumatism, osteoporosis, tendinitis, spine disease); number of health problems among those included in the checklist (headache, back pain, allergy, emotional problem, dizziness, and urinary tract infection); common mental disorder (CMD), assessed using the Self Reported Questionnaire (SRQ-20), being 8 points or more considered CMD present^20^ . We also analyzed the self-assessment of the health status and the duration of the feeling of happiness in the last 15 days, using specific questions from the Medical Outcomes Study 36-Item Short- Form Health Survey (SF-36)^21^ , and life satisfaction, by the question: “Generally, how satisfied are you with your life?”

We also analyzed the associations of self-assessment of sleep quality with variables related to complaints and sleep problems: difficulty in initiating sleep, difficulty in staying asleep, early awakening, snoring, nap during the day (intentional or not), frequency of difficulty in staying awake by day and frequency of good disposition when waking up in the last month, and use of sleeping pills.

The prevalence of poor self-rated sleep was estimated according to the independent variables, and the differences were tested by means of the chi-square test, considering significant those with p-value < 0.05. The prevalence ratios adjusted by sex and age and the respective confidence intervals of 95% were also estimated by Poisson multiple regression. A hierarchical Poisson multiple regression model was developed, in which demographic and socioeconomic variables were entered in the first stage, health behaviors in the second, and indicators of diseases, health problems and welfare in the third stage. Significant variables in one step remained in the model in the following steps. All analyses were performed using the STATA 14.0 statistical software and considering the and considering the weights stemming from the sampling design, non-response rates and post-stratification.

The project of the ISACamp2014/15 survey was approved by the Research Ethics Committee of the School of Medical Sciences of the Universidade Estadual de Campinas (Process 409,714 of 30/09/2013-CAAE 20547513.2.0000.5404).

## RESULTS

Of the households drawn for interviews with adults and elderly, there was loss of 9.2%, being 5.5% by refusal and 3.7% for not finding the resident after more than 3 visits. Of the individuals identified to be interviewed, the percentage of refusal was 21.2% and of other losses, 1.9%. Thus, data from 1,998 individuals aged 20 years and older were analyzed. The study population had a mean age of 43.7 years (95%CI 42.3 – 45.2) and was composed of 52.7% (49,7-55.7%) of women; 9.6% studied less than 4 years and 27.6% studied 12 years or more.

Poor self-rated sleep was reported by 29.1% (95%CI: 26.5-31.7) of adults living in Campinas. Its prevalence was 24.2% (95%CI: 20.4-28.1) in men and 33.5% (95%CI: 30.3-36.7) in women.

Complaints and sleep problems that presented the strongest associations with the poor self-rated sleep were: difficulty in initiating sleep (PR = 4.17) and staying asleep (PR = 4.40), very early awakening (PR = 3.15) and never or almost never woke up cheerful last month (PR = 4.52) ( Table 1 ).

**Table 1:** Prevalence and prevalence ratios of poor self-rated sleep according to characteristics and sleep complaints. Campinas, SP, Brazil, 2014 – 2015.

Characteristics and complaints related to sleep	no.	Prevalence	p	PR adjusted by sex and age	95%CI

		(%)			
Difficulty initiating sleep			< 0.001		
No	1,417	15.6		1	
Yes	580	67.5		4.17	3.26–5.35
Difficulty staying asleep			< 0.001		
No	1,416	15.4		1	
Yes	582	69.9		4.40	3.57–5.41
Early Awakening			< 0.001		
No	1,516	19.7		1	
Yes	467	65.3		3.15	2.64–3.76
Snore			0.05		
No	1,002	26.5		1	
Yes	905	31.7		1.21	1.00–1.46
Sleep duration (in hours)			< 0.001		
≤ 5	86	51.8		2.11	1.59–2.80
6	170	41.1		1.70	1.32–2.18
7–8	953	26.1		1	
≥ 9	778	26.7		0.97	0.8–1.17
Nap during the day			< 0.001		
No	1,692	26.2		1	
Yes, intentionally	105	31.9		1.16	0.99–1.37
Yes, unintentionally	192			1.74	1.26–2.41
Difficulty staying awake during the day in the last month			< 0.001		
Never	1,587			1	
Less 2x per week	210			2.10	1.72–2.56
3x or more per week	192	53.7		2.34	1.88–2.91
Use of sleeping pills					
No	1,790	25.9	< 0.001	1	
Yes	208	67.3		2.30	1.91–2.77
Cheerful when waking up in the last month			< 0.001		
Always	1,097	14.7		1	
Often	249	34.6		2.41	1.86–3.11
Hardly ever or never	194	64.1		4.52	3.57–5.71

Higher prevalence of poor sleep, adjusted for age and sex, was observed in women (PR = 1.36), in individuals with 40 years or more, in those who were not born in Campinas, unemployed and in the segment with the highest number of children (PR = 1.33) ( Table 2 ). Regarding the health behaviors, a significant association was only detected with leisure-time PA. Active individuals present better sleep quality than inactive individuals ( Table 3 ).

**Table 2:** Prevalence and prevalence ratios of poor sleep according to demographic and socioeconomic characteristics. Campinas, SP, Brazil, 2014 – 2015.

Demographic and socioeconomic data	no.	Prevalence	p	PR adjusted by sex and age	95%CI

		%			
Sex			< 0.001		
Male	850	24.2		1	
Female	1.148	33.5		1,36	1,14–1,63
Age Group			0.001		
20–39	549	23.1		1	
40–59	464	34.1		1,46	1,12–1,90
≥ 60	985	34.8		1,50	1,20–1,87
Marital status			0.12		
Married or living with a partner	1.130	29.3		1	
Separate/divorced	180	39.8		1,23	0,89–1,68
Widowed	345	31.1		0,82	0,67–1,00
Single	342	24.2		0,96	0,65–1,41
Place of birth			< 0.001		
Campinas	663	23.3		1	
Other municipality of SP	665	33.7		1,30	1,07–1,57
Other state or other country	670	32.2		1,28	1,02–1,59
Schooling (years)			0.07		
0–3	397	34.6		1	
4–8	732	32.7		1,00	0,79–1,27
9–11	517	27.7		0,94	0,71–1,25
≥ 12	352	24.9		0,86	0,63–1,61
Per capita family income (minimum wage)			0.59		
< 1	671	30.0		1	
1–3	1.046	30.2		1,04	0,87–1,25
> 3	272	26.4		0,91	0,68–1,22
Work			0.001		
Working	941	25.6		1	
Not working	1.056	35.4		1,26	1,03–1,54
Number of children			< 0.001		
0–1	775	24.6		1	
2–3	824	32.4		1,15	0,93–1,43
≥ 4	384	39.1		1,33	1,02–1,74

**Table 3:** Prevalence and prevalence ratios of poor sleep according to health behaviors. Campinas, SP, Brazil, 2014 – 2015.

Health behavior	no.	Prevalence	p	PR adjusted by sex and age	95%CI

		%			
Leisure-time physical activity			0.02		
Active	503	23.8		1	
Insufficiently active	248	26.7		1.03	0.75–1.42
Inactive	1,247	32.1		1.27	1.02–1.58
Weekly intake of alcoholic beverage			0.57		
Do not ingest	1,392	29.5		1	
Ingest once per week or less	501	29.2		1.08	0.86–1.34
Ingest twice or more times per week	105	23.8		0.95	0.65–1.36
Risk consumption of alcoholic beverage			0.48		
No	1,842	29.4		1	
Yes	156	26.4		1.10	0.81–1.49
Smoking			0.48		
Non-smoker	1,386	28.2		1	
Smoker	263	30.9		1.11	0.87–1.41
Former smoker	349	32.0		1.12	0.88–1.42

Prevalence of poor sleep has been growing with the increase in the number of chronic diseases (PR = 1.94 for five or more diseases), the increase in the number of reported health problems (PR = 4.19 for five or more problems), the lower satisfaction with life and the lowest permanence of the feeling of happiness. Poor sleep was significantly more present in individuals with CMD (PR = 2.70) and with regular or poor self-rated health (PR = 2.40) ( Table 4 ).

**Table 4:** Prevalence and prevalence ratios (PR) of poor sleep according to morbidities, state of health and well-being. Campinas, SP, Brazil, 2014 – 2015.

Health and welfare status data	no.	Prevalence	p	PR adjusted by sex and age	95%CI

		%			
Number of chronic diseases			< 0.001		
0	470	19.0		1	
1	398	29.1		1.43	1.08–1.91
2–4	758	32.7		1.56	1.19–2.04
≥ 5	273	43.6		1.94	1.35–2.78
Number of health problems			< 0.001		
0	536	15.1		1	
1	586	26.5		1.75	1.30–2.35
2–4	756	35.7		2.24	1.70–2.96
≥ 5	106	68.6		4.19	3.07–5.7
Common Mental Disorder (SRQ-20)			< 0.001		
No	1,683	23.5		1	
Yes	259	67.1		2.70	2.38–3.08
Self-rated health			< 0.001		
Very good/Good	1,451	22.0		1	
Regular/poor/very poor	540	55.9		2.40	2.03–2.83
Life satisfaction			< 0.001		
Very satisfied	1,314	22.5		1	
More or less satisfied	604	39.5		1.73	1.48–2.03
Not satisfied	59	84.6		3.60	3.06–4.26
Feeling of happiness in the last 15 days			< 0.001		
Always or most of the time	1,451	23.1		1	
Some part of the time	316	43.4		1.74	1.44–2.11
Small part of the time or never	211	59.4		2.42	2.03–3.91

Final regression model confirms the association of sleep quality with sex, age range, place of birth, work (first stage), leisure-time PA (second stage), number of health problems, CMD, self-rated health, and life satisfaction (third stage) ( Table 5 ).

**Table 5:** Hierarchical Poisson regression model of the prevalence of poor self-rated sleep.

Data	1^st^ stage		2^nd^ stage		3^rd^ stage	
		
	PR	95%CI	PR	95%CI	PR	95%CI
Sex						
Male	1		1		1	
Female	1.32	1.10–1.57	1.30	1.10–1.55	1.07	0.89–1.30
Age group						
20-39	1		1		1	
40-59	1.37	1.06–1.75	1.35	1.05–1.72	1.31	1.01–1.71
≥ 60	1.20	0.98–1.49	1.19	0.97–1.46	1.15	0.92–1.45
Place of birth						
Campinas	1		1		1	
Other municipality of SP	1.30	1.07–1.57	1.30	1.07–1.57	1.29	1.06–1.55
Other state or other country	1.27	1.02–1.59	1.26	1.01–1.58	1.23	0.98–1.53
Work						
Working	1		1		1	
Not working	1.22	1.02–1.46	1.27	1.03–1.55	1.08	0.91–1.28
Leisure-time physical activity						
Active			1		1	
Insufficiently active			1.15	0.86–1.54	0.96	0.69–1.35
Inactive			1.28	1.02–1.60	1.01	0.83–1.25
Number of health problems						
None					1	
One					1.44	1.09–1.91
Two to four					1.64	1.23–2.18
Five or more					2.33	1.66–3.29
Self-rated health						
Very good/good					1	
Regular/very bad/bad					1.61	1.32–1.97
CMD						
Absent					1	
Present					1.59	1.34–1.89
Life satisfaction						
Very satisfied					1	
More or less satisfied					1.30	1.09–1.56
Not satisfied					1.67	1.31–2.14

## DISCUSSION

The results show that poor self-rated sleep affects 29.1% of the population of Campinas aged 20 years or older and that the complaints and sleep problems that most influence the individual to classify their own sleep as poor are: difficulty initiating sleep, waking up in the middle of the night, and never or almost never feeling cheerful when waking up. The study found that poor self-rated sleep is significantly more prevalent in women, in people aged 40 years or older, in migrants, in individuals who were not working, in those who do not have leisure-time physical activity, in those with CMD, and in those who evaluate their own health as poor. We also verified that prevalence of poor sleep grows with the increase in the number of health problems and with the level of life dissatisfaction.

A national survey conducted with 47,477 Brazilian workers, who also used a single question to assess the quality of sleep (“How often do you sleep well?”), detected a prevalence of 21% of individuals with poor sleep^12^ . But this population of workers is younger than the one analyzed in our study.

A research developed with adult women in the United States identified that 27% had poor sleep quality^11^ . A survey developed in 10 countries, including Brazil, showed prevalence of individuals who reported not sleeping well between 10.4% in Austria and 32.2% in Belgium; the prevalence in Brazil was 19.2%^9^ . Prevalence higher than that observed in Campinas was found in a study that used the PSQI for rating the sleep quality of Japanese adults and found that 36.4% of women and 30.1% of men reached the cutoff point of poor sleep^22^ . Results differ greatly depending on the study population, instrument used and the means of obtaining information. Variations in the range of 7 to 45% have been found in studies conducted in different countries^12^ .

In relation to sex, as in this survey, the studies found, a higher prevalence of bad sleepers among women^12 , 22^ . Women usually report more health problems than men, use more health services, more frequently exert the role of caregiver of family members, are more attentive to the signs and symptoms of diseases and assume with less embarrassment the patient’s role and the report of disease symptoms. Women may be more susceptible to sleep problems due to hormonal conditions as the changes observed in sleep patterns during pregnancy, menopause and menstrual cycles. Women also present depressive symptoms more frequently than men, and is in charge of family roles and responsibilities that could influence the sleep pattern^11^ .

This study showed a higher prevalence of poor sleep quality in individuals aged 40 years or older, without a growth in prevalence after 60 years ( Table 2 ). The loss of significance of the difference between the segment of 60 years or older and the youngest, in the first step of the regression model ( Table 5 ), stems from the association between the variables age and work. Appleton et al.^23^ analyzed several sleep disorders in Australian adults and did not observe an increase in the prevalence of the surveyed disorders in the segment of 65 years or older. Study by Madrid-Valero et al.^24^ showed worsening of sleep with advancing age, affecting the latency and efficiency of sleep and increasing the prevalence of sleep disorders, but did not find significant differences in the perception of sleep quality.

The occurrence of higher prevalence in individuals not born in Campinas could result from greater exposure to stress, either by living and working conditions, or by family problems that migration processes could imply. Thus, studies that seek to elucidate a possible association between migration and sleep patterns are needed.

Even after adjusting for age and sex, adults and older adults who are not working have a higher prevalence of poor sleep. After analyzing the prevalence of insomnia in adults in Finland, Talala et al.^25^ found a higher prevalence in individuals who were unemployed. Besides the economic insecurity generated by the absence of work affecting the quality of sleep, the working condition itself, when promoting a rhythm of activities during the day, may result in a greater regularity of nocturnal sleep. Verifying that the introduction of the variables of the third step of the regression model (self-rated health, CMD and life satisfaction) causes the association of the working condition with the quality of sleep to disappear and, given the cross-sectional design, it is necessary to consider the possible bidirectionality of the associations. On the one hand, people who do not work can sleep poorly due to worse emotional health and life dissatisfaction, resulting from inactivity at work and, on the other, people in worse physical and emotional health situations (which tend to present worse quality of sleep) would have less condition to stay at work.

Inactive individuals in the leisure time had a higher prevalence of poor sleep, as has also been detected in other studies^12^ , and the association of physical inactivity with depressive feelings, which are strongly associated with low sleep quality, has been consistent in the literature and would partly be responsible for the association between PA and sleep^26^ . However, as this is a cross-sectional study, it is also necessary to consider that individuals with poorer health and well-being (who tend to have poorer quality of sleep) would be less prone or able to practice leisure-time PA.

The concomitant presence of several diseases increases the prevalence of poor sleep, being almost 4.19 times higher in people with five or more health problems. Hayashino et al.^27^ also identified an increase in PSQI scores with the increase in the number of comorbidities. Consistent with this finding, people who consider their own health as poor also evaluate their sleep as being of low quality, as evidenced by other authors^11^ . These findings highlight the importance of health professionals being attentive and working to reduce the risk of the emergence of comorbidities and to monitor the quality of sleep of patients with multiple diseases, considering that bad sleep worsens health quality and increases the risk of mortality^2 , 3^ .

The presence of CMD increased in 61% the prevalence of poor sleep quality, even after adjusting for all the variables included in the model. Disorders are mainly constituted by symptoms of depression or anxiety. The association between life satisfaction and quality of sleep also persisted in the final model. Literature has shown the association between depression, life dissatisfaction and impairment of sleep. Lacruz et al.^28^ , in Germany, studied individuals from a cohort and observed a synergistic effect of depression and low life satisfaction in the prevalence of sleep problems. A meta-analysis that included 21 longitudinal studies confirms insomnia as a risk factor for depression, detecting odds ratio of 2.10^6^ . A longitudinal study developed in China identified the bidirectional relationship between sleep and optimism, in which depressive humor fully intermediates the influence of optimism on sleep quality and partially intermediates the quality of sleep in optimism^29^ . As far as we know, this is the first Brazilian population-based study to evaluate the epidemiological profile, considering a broad set of demographic and health variables, the sleep quality of adults aged 20 years or older. However, it is necessary to consider its limitations in the analysis of the results. The assessment of sleep quality was measured by a single question and not by a complete instrument already validated such as the PSQI. It is noteworthy, however, that other researchers have also used a single question to assess the quality of sleep and obtained results consistent with the use of different questions^9^ . Other authors have also used a single question to assess dissatisfaction with sleep, insufficient sleep^30^ and the presence of insomnia^24^ .

Swinbourne et al.^31^ highlight the strong association between self-rated sleep quality and PSQI scores. Additionally, Ohayon and Zulley^32^ , using a single question to assess overall sleep dissatisfaction, verified that the generated indicator constitutes a better measure of sleep pathology than the symptoms of insomnia alone. In this study, a strong association was found between self-rated sleep quality and sleep complaints. Discussions on the concept and strategies for assessing sleep quality persist and new approaches have been proposed for a more adequate measurement^10 , 33^ .

In the interpretation of our results, we should consider that the data used was obtained by household interviews and was subjected to memory and information biases. The cross-sectional study design, in turn, does not allow characterizing the associations found as causal. The association of sleep quality with work and leisure-time PA, for example, are no longer significant with the introduction of the third stage variables in the model, refers to the consideration of the evidences of bi-directional relations between PA and disiases/well-being, as well as between work and diseases/welfare. Thus, variables of the third stage could be mediators of the effect of PA and work on the quality of sleep or the reverse could be occurring with the variables of the third stage, leading to non-work and non-practice of PA in the leisure time.

## CONCLUSIONS

Based on a representative sample of the population, the study detected a high prevalence of poor sleep quality in the adult population of Campinas and showed that this prevalence were significantly higher in the vulnerable subgroups composed by women, people aged 40 to 59 years, migrants and those who are unemployed. The association of good sleep quality with PA practice should be considered in interventions to promote PA in the leisure time or to increase the quality of sleep. Associations between sleep quality and the presence of chronic diseases and health problems were also found, signaling the need for prevention of comorbidities and attention to sleep quality in patients with multiple diseases. Finally, association of low sleep quality with CMD and life dissatisfaction highlights the importance that should be given to the emotional aspects of patients with poor quality of sleep.
