# Supplementary material for: Quality of sleep, health and well-being in a population-based study
Source: Rev Saude Publica. 2019 Sep 18;53:82. doi: 10.11606/s1518-8787.2019053001067 (PMC6763282; doi:10.11606/s1518-8787.2019053001067)
Supplement: Translation

# Translation: Qualidade do sono, saúde e bem-estar em estudo de base populacional

**Authors:** Marilisa Berti de Azevedo Barros, Margareth Guimarães Lima, Maria Filomena Ceolim, Edilson Zancanella, Tânia Aparecida Marchiori de Oliveira Cardoso

**Affiliations:** I Universidade Estadual de Campinas. Faculdade de Ciências Médicas. Departamento de Saúde de Coletiva. Campinas, São Paulo, Brasil; II Universidade Estadual de Campinas. Faculdade de Enfermagem. Campinas, São Paulo, Brasil; III Universidade Estadual de Campinas. Faculdade de Ciências Médicas. Departamento de Oftalmologia/otorrinolaringologia. Campinas, São Paulo, Brasil; IV Universidade Estadual de Campinas. Faculdade de Ciências Médicas. Departamento de Neurologia. Campinas, São Paulo, Brasil

**Keywords:** Sono, Comorbidade, Fatores de Risco, Fatores Socioeconômicos, Saúde Mental, Inquéritos Epidemiológicos

## Abstract

**OBJETIVO:**

Estimar a prevalência de sono autoavaliado como ruim e identificar os subgrupos da população mais susceptíveis ao problema.

**MÉTODOS:**

Trata-se de estudo transversal, de base populacional, desenvolvido com dados de Inquérito de Saúde conduzido no município de Campinas (ISACamp 2014/2015). Foram analisados dados de amostra representativa de 1.998 indivíduos com 20 anos ou mais de idade. A qualidade autoavaliada do sono foi analisada segundo características sóciodemográficas, morbidades, comportamentos de saúde e sentimentos de bem-estar. Analisou-se também a associação da qualidade do sono com diferentes queixas e características do sono. Foram estimadas razões de prevalências e desenvolvido modelo de regressão múltipla de Poisson, considerando-se nas análises os pesos amostrais.

**RESULTADOS:**

A prevalência de sono autoavaliado como ruim foi 29,1% e mostrou-se significativamente mais elevada nas mulheres, em indivíduos de 40 a 50 anos de idade, migrantes, sem ocupação, fisicamente inativos em contexto de lazer, com transtorno mental comum (RP = 1,59), com maior número de problemas de saúde (RP = 2,33), com saúde autoavaliada como ruim (RP = 1,61) e que manifestavam insatisfação com a vida. Sono ruim esteve fortemente associado com relatos de dificuldade de iniciar o sono (RP = 4,17), de manter o sono (RP = 4,40) e com nunca ou quase nunca se sentir bem-disposto ao acordar (RP = 4,52).

**CONCLUSÕES:**

Os resultados identificam os segmentos da população com má qualidade do sono que merecem maior atenção e destacam a necessidade de avaliar, além da presença de comorbidades, a saúde mental e a presença de sentimentos de bem-estar no processo de cuidado dos pacientes com problemas de sono e no planejamento de intervenções voltadas à promoção de sono saudável.

## INTRODUÇÃO

A qualidade do sono constitui uma das cinco dimensões consideradas relevantes para a avaliação do sono saudável, entendido como um padrão multidimensional de sono-vigília adaptado às demandas individuais, sociais e ambientais e que proporciona bem-estar físico e mental^1^ . Sendo uma necessidade humana básica, o sono é um dos temas emergentes mais relevantes, pois há evidências robustas de que a sua privação e os seus distúrbios afetam processos metabólicos e inflamatórios, com amplos impactos negativos na saúde^2^ .

Especificamente quanto à qualidade do sono, as pesquisas detectaram que a pior qualidade se associa a taxas mais elevadas de mortalidade e a prevalências maiores de síndrome metabólica, diabetes, hipertensão, doença coronariana e depressão^3^ .

Distúrbios do sono, que implicam em baixa qualidade do sono, são também causa frequente de acidentes de trânsito e de trabalho^7^ resultantes da sonolência diurna excessiva que os acompanha. A baixa qualidade do sono acarreta prejuízos nas atividades diárias do indivíduo, afetando o rendimento no trabalho e a qualidade de vida em geral, o que provoca forte impacto social e econômico^2^ .

A avaliação da qualidade do sono vem sendo feita por diferentes instrumentos e em especial pelo *Pittsburgh Sleep Quality Index (PSQI)* ^8^ . Um dos itens desse instrumento questiona, especificamente, sobre a avaliação subjetiva da qualidade do sono e alguns autores têm utilizado uma única pergunta para efetuar essa avaliação^9^ . De maneira análoga, no campo da avaliação da saúde, informação obtida por uma única pergunta sobre a avaliação subjetiva da própria saúde mostrou-se altamente correlacionada com mortalidade, doenças e outros desfechos, passando a constituir um dos indicadores de saúde mais utilizados na atualidade^13 , 14^ .

Pelo forte impacto da qualidade do sono no nível de saúde das populações, torna-se essencial o monitoramento de seus padrões e tendências de forma a identificar os segmentos sociais e demográficos mais vulneráveis, sinalizando as estratégias mais adequadas de controle e tratamento de distúrbios e de iniciativas de promoção de sono saudável. Entretanto, das seis pesquisas brasileiras de base populacional que investigaram características do sono em adultos^15^ , apenas um artigo foi publicado focando especificamente a qualidade do sono de adultos e os fatores associados^17^ , em que foram avaliados apenas indivíduos de 40 anos ou mais.

Nessa perspectiva, considerando-se a carência de estudos sobre qualidade do sono, o constatado crescimento dos problemas e distúrbios do sono^18^ , seu impacto na saúde e na qualidade de vida e a crescente relevância do tema no campo da saúde pública, o objetivo deste estudo foi analisar o perfil epidemiológico do sono autoavaliado como ruim segundo amplo conjunto de características, incluindo sociais e demográficas, estado de saúde, morbidades, comportamentos de saúde e indicadores de bem-estar. A pesquisa tem por objetivo, também, analisar a associação da autopercepção da qualidade do sono com o relato de queixas e características do sono em adultos.

## MÉTODOS

Este estudo transversal de base populacional foi desenvolvido com dados do Inquérito de Saúde do Município de Campinas (ISACamp 2014/15). Esse inquérito foi conduzido em amostra representativa da população residente em domicílios particulares e permanentes da área urbana do município de Campinas, SP, Brasil. O inquérito objetivou analisar as condições de saúde e uso de serviços de saúde de pessoas com 10 anos ou mais e definiu três domínios etários – adolescentes, adultos e idosos – para os quais foram sorteadas amostras independentes.

Foi definido um tamanho amostral mínimo de 1.000 pessoas para adolescentes e idosos e de 1.400 para adultos. Esses tamanhos amostrais permitem estimar uma proporção de 0,50 (máxima variabilidade amostral), com erro de amostragem de até 5 pontos percentuais, com nível de confiança de 95% e considerando um efeito de delineamento de 2. O inquérito foi realizado com amostra por conglomerados, estratificada, e tomada em dois estágios. No primeiro estágio, foram sorteados 70 setores censitários, para os quais foi feita a listagem atualizada dos domicílios. Para obter o tamanho mínimo de indivíduos em cada domínio de idade e tendo por base o número médio de moradores de cada domínio por domicilio, foram sorteados 3.119 domicílios para entrevistar adolescentes, 1.029 para adultos e 3.157 para idosos, considerando taxas de não resposta de 27%, 22% e 20% para os respectivos domínios de idade. Foram entrevistados todos os indivíduos do domínio para o qual a residência havia sido sorteada. Neste estudo, foram analisados apenas os dados de indivíduos com 20 anos ou mais de idade.

O questionário do ISACamp 2014/15 abarca ampla temática de saúde e, neste estudo, foram analisadas informações dos blocos de sono, estilo de vida, morbidades, estado de saúde, saúde emocional, comportamentos de saúde e condições demográficas e socioeconômicas.

As entrevistas domiciliares foram feitas por entrevistadores treinados, que utilizaram software especialmente desenvolvido para esta pesquisa e aplicado por meio de *tablet Samsung Galaxy, model GT-P5200* .

A variável dependente do estudo foi autoavaliação do sono, construída a partir da questão “Como o (a) Sr.(a) avalia a qualidade do seu sono? O(a) Sr.(a) diria que é: excelente/muito bom; bom; regular; ruim; muito ruim?”. Foram agrupadas as alternativas excelente/muito bom e bom, formando a categoria de sono bom; as respostas regular, ruim e muito ruim compuseram a categoria de sono autoavaliado como ruim.

As variáveis independentes analisadas foram:

- Demográficas e socioeconômicas: sexo, idade, situação conjugal, naturalidade, escolaridade em anos de estudo, renda familiar per capita, condição de trabalho e número de moradores.
- Variáveis de comportamentos de saúde: frequência semanal e consumo de risco de bebida alcoólica, avaliado por meio do AUDIT e considerando o ponto de corte de 8 ou mais como positivo^19^ ; tabagismo: fumante, ex-fumante, nunca fumou; e atividade física (AF) em contexto de lazer, obtida com a aplicação do *International Physical Activity Questionnaire* (IPAQ) e usada como base para classificação dos indivíduos em: ativos, quando referiam praticar 150 minutos de AF moderada ou 75 minutos de vigorosa; insuficientemente ativos, quando não atingiam o patamar definido para o ativo; e inativo, quando não praticava AF.
- Morbidade e estado de saúde: número de doenças crônicas entre as referidas como diagnosticadas por profissional de saúde e incluídas em *checklist* (hipertensão, diabetes, angina/infarto, câncer, asma, rinite, sinusite, artrite/reumatismo, osteoporose, tendinite, doença de coluna); número de problemas de saúde entre os incluídos em *checklist* (dor de cabeça, dor nas costas, alergia, problema emocional, tontura e infecção do trato urinário); transtorno mental comum (TMC), avaliado por meio do *Self-Reporting Questionnaire* (SRQ-20), sendo 8 pontos ou mais considerado TMC presente^20^ . Também foram analisadas a autoavaliação do estado de saúde e a duração do sentimento de felicidade nos últimos 15 dias, utilizando perguntas específicas do *The Medical Outcomes Study 36-Item* *Short-Form* *Health Survey* (SF-36)^21^ , e a satisfação com a vida, pela questão: “De um modo geral, quanto o Sr. está satisfeito com sua vida?”.

Também foram analisadas as associações da autoavaliação da qualidade do sono com variáveis relativas a queixas e problemas de sono: dificuldade para iniciar o sono, dificuldade de manutenção do sono, despertar muito cedo, ronco, cochilo durante o dia (intencional ou não), frequência de dificuldade de ficar acordado de dia no último mês, frequência de boa disposição ao acordar no último mês e uso de remédios para dormir.

Foram estimadas as prevalências de sono autoavaliado como ruim segundo as variáveis independentes, e testadas as diferenças por meio do teste qui-quadrado, considerando significativas aquelas com valor de p < 0,05. Também foram estimadas as razões de prevalências ajustadas por sexo e faixa etária e os respectivos intervalos de confiança de 95%, por meio de regressão múltipla de Poisson. Modelo hierárquico de regressão múltipla de Poisson foi desenvolvido, no qual entraram as variáveis demográficas e socioeconômicas na primeira etapa, os comportamentos de saúde na segunda e os indicadores de doenças, de problemas de saúde e de bem-estar na terceira. As variáveis significativas em uma etapa permaneciam no modelo nas etapas seguintes. Todas as análises foram realizadas utilizando o software estatístico STATA 14.0 e levando em conta os pesos do delineamento, da taxa de resposta e da pós-estratificação.

O projeto do inquérito ISACamp2014/15 foi aprovado pelo Comitê de Ética em Pesquisa da Faculdade de Ciências Médicas da Universidade Estadual de Campinas (Parecer 409.714 de 30/09/2013 - CAAE 20547513.2.0000.5404).

## RESULTADOS

Dos domicílios sorteados para entrevistas com adultos e idosos, houve perda de 9,2%, sendo 5,5% por recusa e 3,7% por não encontrar o morador após mais de 3 visitas. Dos indivíduos identificados para serem entrevistados, o percentual de recusa foi 21,2% e de outras perdas, 1,9%. Assim, foram analisados dados de 1.998 indivíduos com 20 anos ou mais. A população estudada apresentou idade média de 43,7 anos (IC95% 42,3–45,2) e foi composta por 52,7% (49,7-55,7%) de mulheres; 9,6% estudaram menos de 4 anos e 27,6% estudaram 12 anos ou mais.

Autoavaliação do sono como ruim foi reportada por 29,1% (IC95%:26,5-31,7) dos adultos residentes em Campinas. Sua prevalência foi de 24,2% (IC95%:20,4-28,1) nos homens e 33,5%(IC95%:30,3-36,7) nas mulheres.

As queixas e problemas do sono que apresentaram as associações mais fortes com a autoclassificação do sono como ruim foram: ter dificuldade de iniciar o sono (RP = 4,17) e de manter o sono (RP = 4,40), despertar muito cedo (RP = 3,15) e nunca ou quase nunca acordar bem-disposto no último mês (RP = 4,52) ( Tabela 1 ).

**Tabela 1:** Prevalência e razões de prevalências de sono autoavaliado como ruim segundo características e queixas de sono. Campinas, SP, Brasil, 2014–2015.

Características e queixas relativas ao sono	n	Prevalência	p	RP ajustada por sexo e idade	IC95%

		(%)			
Dificuldade de início do sono			< 0,001		
Não	1.417	15,6		1	
Sim	580	67,5		4,17	3,26–5,35
Dificuldade de manutenção do sono			< 0,001		
Não	1.416	15,4		1	
Sim	582	69,9		4,40	3,57–5,41
Despertar precoce			< 0,001		
Não	1.516	19,7		1	
Sim	467	65,3		3,15	2,64–3,76
Ronca			0,05		
Não	1.002	26,5		1	
Sim	905	31,7		1,21	1,00–1,46
Duração do sono (em horas)			< 0,001		
≤ 5	86	51,8		2,11	1,59–2,80
6	170	41,1		1,70	1,32–2,18
7–8	953	26,1		1	
≥ 9	778	26,7		0,97	0,8–1,17
Cochila durante o dia			< 0,001		
Não	1.692	26,2		1	
Sim, intencionalmente	105	31,9		1,16	0,99–1,37
Sim, sem intenção	192			1,74	1,26–2,41
Dificuldade de ficar acordado durante o dia no último mês			< 0,001		
Nunca	1.587			1	
Menos que 2x por semana	210			2,10	1,72–2,56
3x ou mais por semana	192	53,7		2,34	1,88–2,91
Uso de remédios para dormir					
Não	1.790	25,9	< 0,001	1	
Sim	208	67,3		2,30	1,91–2,77
Boa disposição ao acordar no último mês			< 0,001		
Sempre	1.097	14,7		1	
Quase sempre	249	34,6		2,41	1,86–3,11
Nunca ou quase nunca	194	64,1		4,52	3,57–5,71

Prevalências mais elevadas de sono ruim, já ajustadas por idade e sexo, foram observadas nas mulheres (RP = 1,36), nos indivíduos com 40 anos ou mais, naqueles não nascidos em Campinas, nos que não estavam trabalhando e no segmento com maior número de filhos (RP = 1,33) ( Tabela 2 ). Em relação aos comportamentos de saúde estudados, associação significativa só foi detectada com AF de lazer. Indivíduos ativos apresentam qualidade do sono melhor que os inativos ( Tabela 3 ).

**Tabela 2:** Prevalência e razões de prevalências de sono ruim segundo características demográficas e socioeconômicas. Campinas, SP, Brasil, 2014–2015.

Variáveis demográficas e socioeconômicas	n	Prevalência	p	RP ajustada por sexo e idade	IC95%

		%			
Sexo			< 0,001		
Masculino	850	24,2		1	
Feminino	1.148	33,5		1,36	1,14–1,63
Faixa Etária			0,001		
20–39	549	23,1		1	
40–59	464	34,1		1,46	1,12–1,90
≥ 60	985	34,8		1,50	1,20–1,87
Situação conjugal			0,12		
Casado/vive junto	1.130	29,3		1	
Separado/divorciado	180	39,8		1,23	0,89–1,68
Viúvo	345	31,1		0,82	0,67–1,00
Solteiro	342	24,2		0,96	0,65–1,41
Naturalidade			< 0,001		
Campinas	663	23,3		1	
Outro município de SP	665	33,7		1,30	1,07–1,57
Outro estado ou outro país	670	32,2		1,28	1,02–1,59
Escolaridade (em anos)			0,07		
0–3	397	34,6		1	
4–8	732	32,7		1,00	0,79–1,27
9–11	517	27,7		0,94	0,71–1,25
≥ 12	352	24,9		0,86	0,63–1,61
Renda familiar per capita (em salários mínimos)			0,59		
< 1	671	30,0		1	
1–3	1.046	30,2		1,04	0,87–1,25
> 3	272	26,4		0,91	0,68–1,22
Trabalho			0,001		
Trabalha	941	25,6		1	
Não trabalha	1.056	35,4		1,26	1,03–1,54
Número de filhos			< 0,001		
0–1	775	24,6		1	
2–3	824	32,4		1,15	0,93–1,43
≥ 4	384	39,1		1,33	1,02–1,74

**Tabela 3:** Prevalência e razões de prevalências de sono ruim segundo comportamentos de saúde. Campinas, SP, Brasil, 2014–2015.

Comportamentos de saúde	n	Prevalência	p	RP ajustada por sexo e idade	IC95%

		%			
Atividade física no lazer			0,02		
Ativo	503	23,8		1	
Insuficientemente ativo	248	26,7		1,03	0,75–1,42
Inativo	1.247	32,1		1,27	1,02–1,58
Frequência semanal de ingestão de bebida alcoólica			0,57		
Não ingere	1.392	29,5		1	
Ingere 1 vez por semana menos	501	29,2		1,08	0,86–1,34
Ingere 2 vezes ou mais semana	105	23,8		0,95	0,65–1,36
Consumo de risco de bebida alcoólica			0,48		
Não	1.842	29,4		1	
Sim	156	26,4		1,10	0,81–1,49
Tabagismo			0,48		
Não fumante	1.386	28,2		1	
Fumante	263	30,9		1,11	0,87–1,41
Ex-fumante	349	32,0		1,12	0,88–1,42

A prevalência de sono ruim mostrou-se crescente com o aumento do número de doenças crônicas (RP = 1,94 para cinco ou mais doenças), o aumento do número de problemas de saúde relatados (RP = 4,19 para cinco ou mais problemas), a menor satisfação com a vida e a menor permanência do sentimento de felicidade. Sono ruim foi significativamente mais presente nos indivíduos com TMC (RP = 2,70) e com saúde autoavaliada como regular ou ruim (RP = 2,40) ( Tabela 4 ).

**Tabela 4:** Prevalência e razões de prevalências (RP) de sono ruim segundo morbidades, estado de saúde e bem-estar. Campinas, SP, Brasil, 2014–2015.

Variáveis de estado de saúde e bem-estar	n	Prevalência	p	RP ajustada por sexo e idade	IC95%

		%			
Número de doenças crônicas			< 0,001		
0	470	19,0		1	
1	398	29,1		1,43	1,08–1,91
2–4	758	32,7		1,56	1,19–2,04
≥ 5	273	43,6		1,94	1,35–2,78
Número de problemas de saúde			< 0,001		
0	536	15,1		1	
1	586	26,5		1,75	1,30–2,35
2–4	756	35,7		2,24	1,70–2,96
≥ 5	106	68,6		4,19	3,07–5,7
Transtorno Mental Comum (SRQ-20)			< 0,001		
Não	1.683	23,5		1	
Sim	259	67,1		2,70	2,38–3,08
Autoavaliação da saúde			< 0,001		
Muito boa/Boa	1.451	22,0		1	
Regular/Ruim/Muito ruim	540	55,9		2,40	2,03–2,83
Satisfação com a vida			< 0,001		
Muito satisfeito	1.314	22,5		1	
Mais ou menos satisfeito	604	39,5		1,73	1,48–2,03
Nada satisfeito	59	84,6		3,60	3,06–4,26
Sentimento de felicidade nos últimos 15 dias			< 0,001		
Sempre ou a maior parte do tempo	1.451	23,1		1	
Alguma parte do tempo	316	43,4		1,74	1,44–2,11
Pequena parte do tempo ou nunca	211	59,4		2,42	2,03–3,91

O modelo de regressão final confirma a associação da qualidade do sono com sexo, faixa etária, naturalidade, trabalho (primeiro estágio), AF no lazer (segundo estágio), número de problemas de saúde, TMC, autoavaliação da saúde e satisfação com a vida (terceiro estágio) ( Tabela 5 ).

**Tabela 5:** Modelo hierárquico de regressão de Poisson da prevalência de sono autoavaliado como ruim.

Variáveis	1º estágio		2º estágio		3º estágio	
		
	RP	IC95%	RP	IC95%	RP	IC95%
Sexo						
Masculino	1		1		1	
Feminino	1,32	1,10–1,57	1,30	1,10–1,55	1,07	0,89–1,30
Faixa Etária						
2039	1		1		1	
4059	1,37	1,06–1,75	1,35	1,05–1,72	1,31	1,01–1,71
≥ 60	1,20	0,98–1,49	1,19	0,97–1,46	1,15	0,92–1,45
Naturalidade						
Campinas	1		1		1	
Outro município de SP	1,30	1,07–1,57	1,30	1,07–1,57	1,29	1,06–1,55
Outro estado ou outro país	1,27	1,02–1,59	1,26	1,01–1,58	1,23	0,98–1,53
Trabalho						
Trabalha	1		1		1	
Não trabalha	1,22	1,02–1,46	1,27	1,03–1,55	1,08	0,91–1,28
Atividade física no lazer						
Ativo			1		1	
Insuficientemente ativo			1,15	0,86–1,54	0,96	0,69–1,35
Inativo			1,28	1,02–1,60	1,01	0,83–1,25
Número de problemas de saúde						
Nenhum					1	
Um					1,44	1,09–1,91
Dois a quatro					1,64	1,23–2,18
Cinco ou mais					2,33	1,66–3,29
Autoavaliação da saúde						
Muito boa/Boa					1	
Regular/Ruim/Muito ruim					1,61	1,32–1,97
TMC						
Ausente					1	
Presente					1,59	1,34–1,89
Satisfação com a vida						
Muito satisfeito					1	
Mais ou menos satisfeito					1,30	1,09–1,56
Nada satisfeito					1,67	1,31–2,14

## DISCUSSÃO

Os resultados mostram que o sono autoavaliado como ruim atinge 29,1% da população de Campinas com 20 anos ou mais de idade e que as queixas e problemas de sono que mais influenciam o indivíduo a classificar o próprio sono como ruim são: dificuldade de iniciar o sono, acordar no meio da noite e nunca ou quase nunca se sentir bem-disposto ao acordar. O estudo constatou que sono autoclassificado como ruim é significativamente mais prevalente nas mulheres, nas pessoas com 40 anos ou mais, nos migrantes, nos indivíduos que não estavam trabalhando, naqueles que não são fisicamente ativos em contexto de lazer, nos que apresentam TMC e nos que avaliam a própria saúde como ruim. Verificou-se, também, que a prevalência de sono ruim cresce com o aumento do número de problemas de saúde e com o nível de insatisfação com a vida.

Em pesquisa nacional realizada com 47.477 trabalhadores brasileiros, que utilizou também uma única pergunta para avaliar a qualidade do sono (“Com que frequência você avalia que dorme bem?”), foi detectada uma prevalência de 21% de indivíduos com sono ruim^12^ . Mas essa população de trabalhadores é mais jovem que a analisada no presente estudo. Pesquisa desenvolvida com mulheres adultas nos Estados Unidos identificou que 27% apresentavam má qualidade do sono^11^ . Inquérito desenvolvido em 10 países, incluindo o Brasil, mostrou prevalências de indivíduos que relatavam que não dormiam bem entre 10,4% na Áustria e 32,2% na Bélgica; a prevalência no Brasil foi de 19,2%^9^ . Prevalência superior à verificada em Campinas foi constatada em estudo realizado em população de adultos japoneses, que utilizou como instrumento de avaliação da qualidade do sono o PSQI e verificou que 36,4% das mulheres e 30,1% dos homens atingiam o ponto de corte de sono ruim^22^ . Os resultados diferem muito entre as pesquisas dependendo da população estudada, do instrumento utilizado e do meio de obtenção das informações. Variações na faixa de 7 a 45% têm sido constatadas em estudos realizados em diferentes países^12^ .

Em relação a gênero, assim como na presente pesquisa, os estudos constatam, em geral, maior prevalência de maus dormidores entre as mulheres^12 , 22^ . As mulheres usualmente relatam mais problemas de saúde que os homens, utilizam mais os serviços de saúde, exercem com maior frequência o papel de cuidadora dos doentes da família, são mais atentas aos sinais e sintomas das doenças e assumem com menor constrangimento o papel de doente e o relato de sintomas das doenças. As mulheres podem ser mais susceptíveis a problemas de sono devido a alterações hormonais, o que se evidencia com as mudanças de padrão de sono na gravidez, na menopausa e nos ciclos menstruais. Também apresentam sintomas depressivos com maior frequência que os homens, além de papeis e responsabilidades que socialmente lhe são atribuídos e que influenciam no padrão do sono^11^ .

O presente estudo apontou maior prevalência de qualidade de sono ruim nos indivíduos com 40 anos ou mais, sem evidenciar crescimento da prevalência após os 60 anos ( Tabela 2 ). A perda de significância da diferença entre o segmento de 60 anos ou mais e o mais jovem, na primeira etapa do modelo de regressão ( Tabela 5 ), decorre da associação entre as variáveis idade e trabalho. Appleton et al.^23^ analisaram vários distúrbios do sono em adultos australianos e também não verificaram aumento de prevalência dos distúrbios pesquisados no segmento de 65 anos ou mais. Pesquisa de Madrid-Valero et al.^24^ mostrou piora do sono com o avanço da idade, afetando a latência e eficiência do sono e aumentando a prevalência de distúrbios do sono, mas não encontrou diferenças significativas quanto à percepção da qualidade do sono.

A ocorrência de maior prevalência nos indivíduos não naturais do município de Campinas poderia decorrer de maior exposição a estresse, seja por condições de vida e de trabalho, seja por problemas familiares que processos de migração poderiam acarretar. Considera-se que são necessários estudos que busquem elucidar uma possível associação entre migração e padrões de sono.

Mesmo após ajuste para idade e sexo, adultos e idosos que não estão trabalhando apresentam maior prevalência de sono ruim. Analisando a prevalência de insônia em adultos na Finlândia, Talala et al.^25^ encontraram maior prevalência nos indivíduos que não estavam exercendo alguma ocupação. Além de a insegurança econômica gerada na ausência de trabalho poder repercutir sobre a qualidade do sono, a condição de trabalho em si, ao imprimir um ritmo de atividades durante o dia, pode redundar em uma maior regularidade do sono noturno. Verificando que a introdução de cada uma das variáveis da terceira etapa do modelo de regressão (autoavaliação da saúde, TMC e satisfação com a vida) faz desaparecer a associação da condição de trabalho com a qualidade do sono e dado o corte transversal do estudo, é preciso considerar a possível bidirecionalidade das associações. Por um lado, pessoas que não trabalham podem dormir mal devido à pior saúde emocional e insatisfação com a vida, decorrentes da inatividade no trabalho e, por outro, pessoas em pior situação de saúde física e emocional (que tendem a apresentar pior qualidade do sono) teriam menor condição de se manter no trabalho.

Indivíduos inativos em contexto de lazer apresentaram maior prevalência de sono ruim, como também tem sido detectado em outros estudos^12^ , e a associação da inatividade física com sentimentos depressivos, que são fortemente associados à baixa qualidade do sono, tem sido consistente na literatura e seria, em parte, responsável pela associação entre AF e sono^26^ . Porém, considerando-se o corte transversal do presente estudo, é preciso aventar também que indivíduos em pior condição de saúde e bem-estar (que tendem a apresentar pior qualidade do sono) estariam menos propensos ou aptos a praticar AF de lazer.

A presença concomitante de várias doenças aumenta a prevalência de sono ruim, chegando a ser quase 4,19 vezes mais elevada nas pessoas com cinco ou mais problemas de saúde. Hayashino et al.^27^ também identificaram aumento dos escores do PSQI com o aumento do número de comorbidades. Coerente com esse achado, pessoas que consideram a própria saúde como ruim também avaliam seu sono como sendo de baixa qualidade, assim como constatado por outros autores^11^ . Esses achados destacam a importância de os profissionais de saúde estarem atentos e atuando no sentido de reduzir o risco do surgimento de comorbidades e de monitorarem a qualidade do sono dos pacientes com múltiplas doenças, considerando que sono ruim piora ainda mais a qualidade de saúde e aumenta o risco de mortalidade^2 , 3^ .

Detectou-se que a presença de TMC aumentou em 61% a prevalência de má qualidade do sono, mesmo após ajuste por todas as variáveis incluídas no modelo. Os transtornos são constituídos principalmente por sintomas de depressão ou ansiedade. A associação entre satisfação com a vida e qualidade do sono também persistiu no modelo final. A literatura tem mostrado a associação entre depressão, insatisfação com a vida e prejuízo do sono. Lacruz et al.^28^ , na Alemanha, estudaram indivíduos de uma coorte e observaram um efeito sinérgico de depressão e baixa satisfação com a vida na prevalência de problemas do sono. Uma meta-análise que incluiu 21 estudos longitudinais confirma a insônia como fator de risco para depressão, detectando razão de *odds* de 2,10^6^ . Estudo longitudinal desenvolvido na China identificou o relacionamento bidirecional entre sono e otimismo, em que o humor depressivo intermedia totalmente a influência do otimismo na qualidade do sono e intermedia parcialmente a qualidade do sono no otimismo^29^ .

Ressalte-se que no nosso conhecimento este é o primeiro estudo brasileiro de base populacional a avaliar o perfil epidemiológico, considerando amplo conjunto de variáveis demográficas e de saúde, da qualidade do sono de população de adultos com 20 anos ou mais. Entretanto, é preciso considerar suas limitações na análise dos resultados. A avaliação da qualidade do sono foi aferida por uma única pergunta e não por um instrumento completo já validado como o PSQI. Ressalta-se, porém, que outros pesquisadores também têm utilizado uma única pergunta para avaliar a qualidade do sono e obtido resultados consistentes com o uso de diferentes questões^9^ . Outros autores têm utilizado também uma única pergunta para avaliar insatisfação com o sono, sono insuficiente^30^ e a presença de insônia^24^ . Swinbourne et al.^31^ destacam a forte associação entre a autoavaliação da qualidade do sono e os escores do PSQI. Adicionalmente, Ohayon e Zulley^32^ , utilizando uma única pergunta para avaliar a insatisfação global com o sono, verificaram que o indicador gerado constitui melhor indicador de patologia de sono do que os sintomas de insônia sozinhos. No presente estudo, observou-se fortíssima associação da autoavaliação da qualidade do sono com as queixas de sono. As discussões sobre o conceito e as estratégias de avaliação da qualidade do sono persistem e novos enfoques têm sido propostos para uma mensuração mais adequada^10 , 33^ .

Na interpretação dos resultados deste estudo, deve-se também considerar que as informações utilizadas foram obtidas por entrevista domiciliar e sujeitas a vieses de memória e informação. O corte transversal do estudo, por sua vez, impossibilita a caracterização das associações encontradas como causais. A associação da qualidade do sono com trabalho e AF de lazer, por exemplo, que deixam de ser significativas com a introdução das variáveis da terceira etapa do modelo, remete à consideração sobre as evidências de relações bidirecionais entre AF e doenças/bem-estar, assim como entre trabalho e doenças/bem-estar. Dessa forma, as variáveis da terceira etapa poderiam ser mediadoras do efeito de AF e trabalho sobre a qualidade do sono ou o reverso poderia estar ocorrendo com as variáveis da terceira etapa, levando ao não trabalho e à não prática de AF em contexto de lazer.

## CONCLUSÕES

Com base em amostra representativa da população, o estudo detectou a elevada prevalência de sono de má qualidade da população adulta de Campinas e constatou que essas prevalências eram mais elevadas nos subgrupos compostos por mulheres, pessoas com 40 a 59 anos, migrantes e aquelas que não trabalham. A associação da boa qualidade do sono com a prática de AF deve ser considerada em intervenções para promover AF no lazer ou para melhorar a qualidade do sono. Também foram verificadas associações entre a qualidade do sono e a presença de doenças crônicas e problemas de saúde, sinalizando a necessidade de prevenção de comorbidades e de atenção à qualidade do sono de pacientes com múltiplas doenças. Por fim, a associação da baixa qualidade do sono com TMC e com sentimento de insatisfação com a vida destaca a relevância da atenção que deve ser dada aos aspectos emocionais de pacientes com má qualidade do sono.
